# Complementary Functions of Plant AP Endonucleases and AP Lyases during DNA Repair of Abasic Sites Arising from C:G Base Pairs

**DOI:** 10.3390/ijms22168763

**Published:** 2021-08-16

**Authors:** Marina Jordano-Raya, Cristina Beltrán-Melero, M. Dolores Moreno-Recio, M. Isabel Martínez-Macías, Rafael R. Ariza, Teresa Roldán-Arjona, Dolores Córdoba-Cañero

**Affiliations:** 1 Department of Genetics, University of Córdoba, 14071 Córdoba, Spain; b52joram@uco.es (M.J.-R.); cristinitabeltran95@gmail.com (C.B.-M.); b42morem@uco.es (M.D.M.-R.); q92mamam@uco.es (M.I.M.-M.); ge1roarr@uco.es (R.R.A.); ge2roarm@uco.es (T.R.-A.); 2 Maimónides Biomedical Research Institute of Córdoba (IMIBIC), 14004 Córdoba, Spain; 3 Reina Sofía University Hospital, University of Córdoba, 14004 Córdoba, Spain

**Keywords:** abasic sites, AP endonucleases, AP lyases, base excision repair, *Arabidopsis*

## Abstract

Abasic (apurinic/apyrimidinic, AP) sites are ubiquitous DNA lesions arising from spontaneous base loss and excision of damaged bases. They may be processed either by AP endonucleases or AP lyases, but the relative roles of these two classes of enzymes are not well understood. We hypothesized that endonucleases and lyases may be differentially influenced by the sequence surrounding the AP site and/or the identity of the orphan base. To test this idea, we analysed the activity of plant and human AP endonucleases and AP lyases on DNA substrates containing an abasic site opposite either G or C in different sequence contexts. AP sites opposite G are common intermediates during the repair of deaminated cytosines, whereas AP sites opposite C frequently arise from oxidized guanines. We found that the major *Arabidopsis* AP endonuclease (ARP) exhibited a higher efficiency on AP sites opposite G. In contrast, the main plant AP lyase (FPG) showed a greater preference for AP sites opposite C. The major human AP endonuclease (APE1) preferred G as the orphan base, but only in some sequence contexts. We propose that plant AP endonucleases and AP lyases play complementary DNA repair functions on abasic sites arising at C:G pairs, neutralizing the potential mutagenic consequences of C deamination and G oxidation, respectively.

## 1. Introduction

Abasic (apurinic/apyrimidinic, AP) sites are inescapable DNA lesions arising by spontaneous hydrolysis of the N-glycosylic bond between intact nucleobases and deoxyribose [1]. Spontaneous base release is additionally facilitated by some alterations induced by genotoxic compounds. For example, methylation of guanine (N7-methylguanine, N7-meG) results in weakening of the N-glycosylic bond and a marked increase in base loss [2,3,4]. AP sites are also enzymatically generated as intermediates during the Base Excision Repair (BER) pathway, which is initiated by DNA glycosylases that catalyse the excision of modified bases from DNA [5,6,7,8]. It has been estimated that mammalian cells have steady-state levels of 50,000–200,000 AP sites per genome under physiological conditions [9]. AP sites exist as an equilibrium mixture of hemiacetals of the closed furanose form, but approximately 1% is present as the ring-opened aldehyde species which is prone to spontaneous hydrolysis and may generate single-strand breaks (SSBs) [10,11]. Unrepaired AP sites are cytotoxic since they block DNA replication and transcription. DNA replication blockage may be avoided through translesion DNA synthesis across the AP site, which usually results in mutations [12,13].

AP sites are generally repaired through the BER pathway [6,14,15], although other DNA repair routes, such as Nucleotide Excision Repair (NER) may contribute as backup mechanisms [16]. The repair of an AP site through BER requires the removal of the deoxyribose phosphate moiety from DNA to allow the insertion of an intact deoxyribonucleotide. Such removal may be initiated by two distinct classes of enzymes: AP endonucleases and AP lyases [17]. AP endonucleases cleave the phosphodiester bond at the 5′ side of the AP site, generating a strand break with a free 3′-OH terminus and a blocking 5′-deoxyribose phosphate (5′-dRP) end. In contrast, AP lyases perform the incision at the 3′ side of the AP site by cleaving the sugar moiety through a β-elimination mechanism that generates a blocking 3′-phosphor-α,β-unsaturated aldehyde (3′-PUA) and a free 5′-P terminus. A subset of AP lyases perform a β,δ-elimination; thus, generating a blocking 3′-P end. The 5′- and 3′-blocked ends generated by the incision activity of AP endonucleases and AP lyases, respectively, are removed by downstream enzymes before gap filling and ligation achieve a full repair [7,8].

AP lyase activity is usually found in the so-called bifunctional DNA glycosylases, which are able to incise the AP site generated by their own N-glycosylase activity. The remaining DNA glycosylases lack such capability and are called monofunctional [18]. The biological relevance of the AP lyase activity of bifunctional DNA glycosylases (also termed DNA glycosylases/AP lyases) is not well understood. In particular, it has been a long-standing question whether such enzymes are able to process in vivo AP sites not generated by their own DNA glycosylase activity [19]. Based on evidence chiefly obtained in mammalian cells, it has been generally accepted that, in vivo, the vast majority of AP sites, either from spontaneous or enzymatic origin, are repaired by AP endonucleases [20]. 

However, studies in non-mammalian systems point to a physiological role for AP lyases in removing AP sites arising independently of N-glycosylase activity. In *S. pombe*, for example, most abasic sites are incised by the AP lyase activity of the bifunctional DNA glycosylase Nthp1, generating nicks with 3′-PUA ends that are converted to 3′-OH by the phosphodiesterase activity of Apn2, the major AP endonuclease in fission yeast [21,22]. Results obtained in *S. cerevisiae* suggest an analogous scenario, in which the AP lyase activity of Nthp1 homologs Ntg1 and Ntg2 acts upstream of AP endonucleases Apn1 and Apn2 during repair of AP sites [23]. Thus, AP endonucleases in yeast may predominantly function in the removal of 3′-blocks generated by AP lyases. Interestingly, it has been recently reported that the phosphodiesterase activity of human APE1 plays a relevant role in processing 3′-PUA ends generated by the lyase activity of NTHL1 in nucleosomal, but not in naked, DNA [24]. Additionally, it has been shown that the bifunctional DNA glycosylase NEIL2, which is upregulated in the breast cancer cell line Hs578T, outcompetes APE1 at AP sites and sensitizes breast cancer cells to APOBEC3 deaminase-mediated mutations [25].

In plants, we recently reported that FPG, the major AP lyase of *Arabidopsis thaliana*, has a relevant biological role in the repair of AP sites generated by the spontaneous release of N7-meG [26]. Such AP sites are very poor substrates for ARP, the major AP endonuclease in *Arabidopsis*, but are efficiently incised by the AP lyase activity of FPG, a β, δ-elimination catalyst. The blocking 3′-P ends generated by FPG are processed by the DNA 3′-phosphatase ZDP, allowing to complete repair in an AP endonuclease-independent pathway [26].

In *Arabidopsis*, both ARP and FPG incise enzymatically generated AP sites, but the factors implicated in the choice between endonuclease- or lyase-initiated repair remain unknown. For some BER enzymes targeting the same lesion, for example, different uracil DNA glycosylases, two important specificity factors are the flanking sequence and the identity of the opposite base on the complementary strand. Thus, in *Arabidopsis,* both UNG and MBD4L DNA glycosylases excise uracil, which commonly arises from spontaneous C deamination [1]. However, whereas UNG displays flexibility for the opposite base and the flanking sequence [27], MBD4L only excises U when opposite G and, additionally, shows a strong preference for a DNA sequence context (5′-CG-3′) with a high probability of cytosine methylation [28]. We proposed that MBD4L has evolved to specifically counteract C and 5-meC deamination at CG sequences, where most plant DNA methylation is found [28]. Additionally, the *Arabidopsis* 5-methylcytosine (5-meC) DNA glycosylase ROS1 efficiently excises T (=5-methyluracil, 5-meU) but only at T:G mismatches, and also displays a strong preference for a CG sequence context [29,30]. Therefore, the specificity of some BER enzymes is dictated by both the opposite base and the methylation probability of the sequence context. Based on these observations and our previous results with ARP and FPG [26], we hypothesized that the probability of methylation at the sequence flanking the AP site and/or the orphan base on the opposite DNA strand may influence the probability that an abasic site is processed either by an AP endonuclease or an AP lyase. 

In this work, we analysed the activity of plant and human AP endonucleases and AP lyases on DNA substrates containing an abasic site opposite either G or C in different sequence contexts with different methylation probabilities. AP sites opposite G are common intermediates during the repair of deaminated C or 5-meC, whereas AP sites opposite C arise after spontaneous N7-meG depurination or during the repair of oxidized G [7]. We found that in all tested sequence contexts, *Arabidopsis* ARP endonuclease displayed a significantly higher activity on AP sites opposite G. In contrast, FPG AP lyase showed a preference for AP sites opposite C. The major human AP endonuclease (APE1) preferred G as the orphan base in some sequence contexts, whereas in the AP lyase activity detected in human cells extracts the opposite base dependence was different for β- and β,δ-elimination catalysts. Our results suggest that plant AP endonucleases and AP lyases perform complementary functions in the maintenance of C:G pairs, counteracting the potential mutagenic consequences of C deamination and G oxidation, respectively.

## 2. Results

### 2.1. Design of DNA Substrates

**Figure 1:**
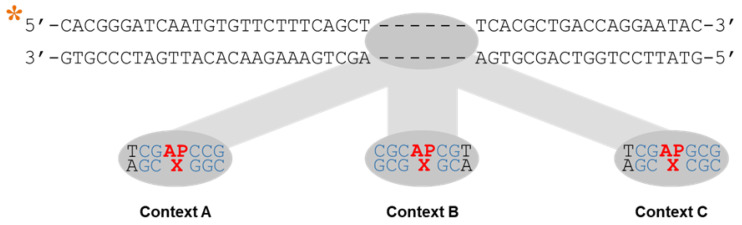
Oligonucleotide duplexes with three different sequence contexts and either G or C opposite the AP site used as DNA substrates. Fluorescein labelling at the 5′ end of the upper strand is indicated by an asterisk. X indicates G or C.

Genomic studies performed in *Arabidopsis* determined the relationship between sequence context and probability of methylation in the three methylation contexts existing in plants, CG, CHG and CHH [31,32]. Based on such data, we designed 5′-fluorescein-labelled 51-mer oligonucleotides with a single uracil residue in three sequence contexts with different probabilities of methylation (Figure 1). Since we were also interested in analysing the effect of the orphan base at AP sites arising from C:G pairs, we designed complementary oligonucleotides with C or G opposite the uracil. Next, the abasic site was generated by incubation with *Escherichia coli* Uracil DNA Glycosylase (UDG) (see Materials and Methods). Depending on the opposite base, the six different DNA substrates present different probabilities of DNA methylation at the orphan cytosine (when the opposite base is C) or the lost cytosine (when the opposite base is G) (Supplementary Table S1).

### 2.2. The Main Arabidopsis AP Endonuclease, ARP, Prefers G to C as the Base Opposite the Abasic Site

**Figure 2:**
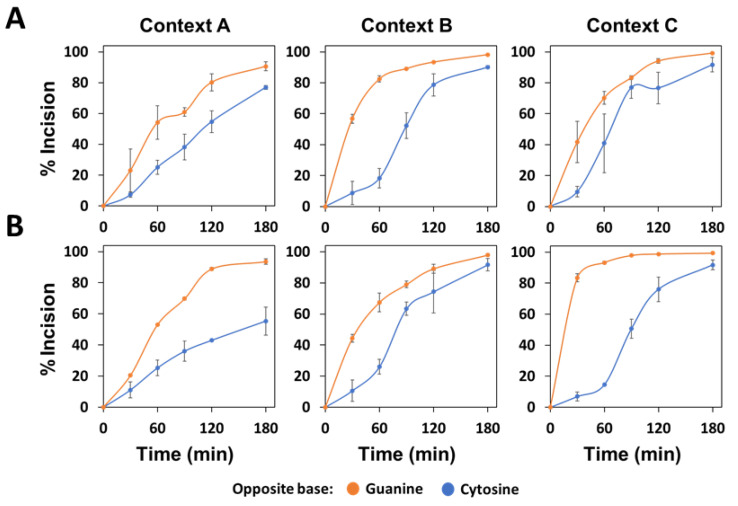
Effect of flanking sequence context and orphan base on the AP endonuclease activity of recombinant *Arabidopsis* ARP. His-ARP protein (10 nM) was incubated at 37 °C with 40 nM (**A**) or 80 nM (**B**) DNA substrates (contexts A, B or C) containing an AP site opposite guanine (orange) or cytosine (blue). After stabilization with NaBH_4_, reaction products were separated by denaturing PAGE, detected by fluorescence scanning and quantified. Data are the mean and standard error from three independent experiments.

To determine whether the activity of the main *Arabidopsis* AP endonuclease is influenced by a context sequence with a different preference to be methylated and/or by the base opposite the abasic site, we performed incision assays with recombinant ARP protein and the six different DNA substrates described above. The results obtained show a preference of recombinant ARP in processing AP sites opposite G in comparison to AP:C sites in all three sequence contexts (Figure 2). The preference of ARP for AP:G targets was most evident with context C and the higher substrate concentration, with more than 80% of DNA being processed when the AP site was opposite guanine, but less than 10% when the orphan base was cytosine (Figure 2B).

In general, ARP showed a higher activity on AP sites located at B or C contexts, being the A context processed with a lower efficiency (Figure 2). In reactions with 80 nM of AP:G substrates (Figure 2B), a preference of the enzyme for sequence context C was observed, reaching 80% of the processed substrate in 30 min, whereas only 20% or 40% substrate was processed with substrates with contexts A or B, respectively. With AP:C targets, lower incision efficiencies in the A context were also observed, particularly at the higher DNA concentration. Thus, at 180 min and 80 nM DNA, only 55% of substrate with context A was processed, compared to 91% for contexts B or C.

**Figure 3:**
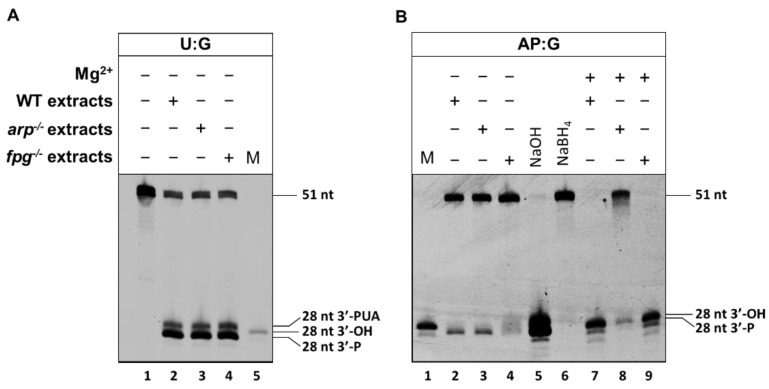
Incision assay for characterization of *Arabidopsis* WT, *arp^−/−^* and *fpg^−/−^* cell extracts. (**A**) Uracil DNA glycosylase activity. Cell extracts from WT, *arp^−/−^* and *fpg^−/−^* lines (25 µg) were incubated for 3 h at 37 °C in the presence of 20 nM of DNA substrate containing an uracil. Reaction products were separated by denaturing PAGE and detected by fluorescence scanning. M, 28 size marker with 3′-OH. (**B**) AP lyase and AP endonuclease activity. WT, *arp^−/−^* and *fpg^−/−^* cell extracts (25 µg) were incubated for 3 h at 37 °C in the presence of 20 nM of DNA substrate containing an AP site opposite G, either in the presence or in the absence of 2 mM Mg^2+^. After stabilization with NaBH_4_, reaction products were separated and detected as indicated above.

We next analysed the effect of the sequence context and orphan base on the native ARP activity detected in plant whole-cell extracts. To exclude AP lyase activity, we used *fpg^−/−^* mutant plants. The cell extract quality and DNA repair competence were previously verified by measuring uracil DNA glycosylase activity in comparison with WT extracts on a DNA duplex containing uracil (Figure 3A). We then examined the level of AP endonuclease and AP lyase activities in cell extracts from WT, *arp*^−/−^ and *fpg^−/−^* plants on a DNA substrate containing an AP site (Figure 3B). The Mg^2+^-dependent AP incision activity was lost in *arp*^−/−^ mutants. In contrast, AP incision levels obtained with *fpg^−/−^* extracts were comparable to those of WT extracts, but only in the presence of Mg^2+^; thus, demonstrating that the only AP processing capacity detected in *fpg^−/−^* extracts was AP endonuclease activity from ARP. 

**Figure 4:**
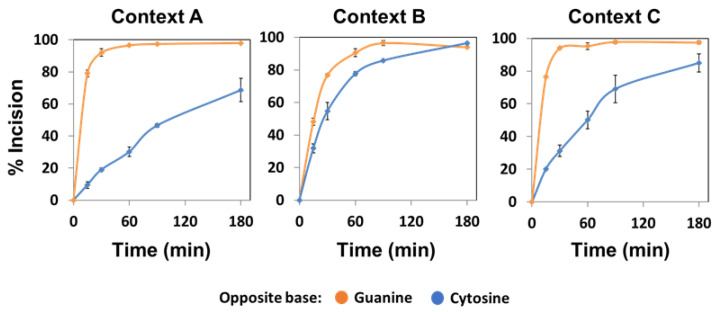
Effect of sequence context and base opposite the abasic site on the AP endonuclease activity of native ARP. Cell extracts from *fpg^−/−^* plants (10 μg) were incubated at 37 °C with 20 nM DNA substrates (contexts A, B or C) containing an AP site opposite guanine (orange) or cytosine (blue). After stabilization with NaBH_4_, the reaction products were separated by denaturing PAGE, detected by fluorescence scanning and quantified. Data are the mean and standard error from three independent experiments.

When we analysed the AP incision activity of native ARP on the different DNA substrates, we also observed a clear preference for AP sites opposite guanine (Figure 4). In contrast to recombinant ARP, curves obtained with the native enzyme were rectangular hyperbolas that could be fitted to the equation [Product] = P_max_ [1 − exp^(−kt)^]; thus, allowing the calculation of kinetic parameters (see Materials and Methods). The relative processing efficiency (E_rel_) of native ARP on AP:G was significantly higher than on AP:C in all three sequence contexts (Supplementary Table S2). However, the preference for G as the orphan base was less noticeable in context B. As a result, for AP sites opposite guanine, a lower E_rel_ was detected in context B (1.38 ± 0.02) compared to contexts A and C (3.10 ± 0.22 and 2.91 ± 0.02, respectively). Conversely, for AP sites opposite cytosine, the E_rel_ on context B was higher (0.75 ± 0.01) than that observed in contexts A and C (0.19 ± 0.02 and 0.38 ± 0.02, respectively). Altogether, these results indicate that ARP, the main AP endonuclease from *Arabidopsis*, displays a significant preference for abasic sites opposite guanine, and that its enzymatic activity is modulated by the specific sequence flanking the lesion.

### 2.3. The Main Arabidopsis AP Lyase, FPG, Prefers G to C as the Base Opposite the Abasic Site

**Figure 5:**
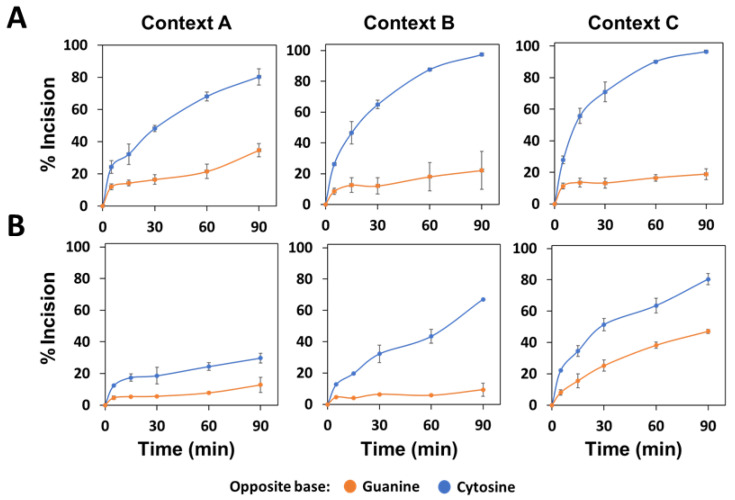
Effect of sequence context and base opposite the abasic site on the AP lyase activity of recombinant FPG. His-FPG protein (2 nM) was incubated at 37 °C with 20 nM (**A**) or 40 nM (**B**) of DNA substrates (contexts A, B or C) containing an AP site opposite guanine (orange) or cytosine (blue). After stabilization with NaBH_4_, reaction products were separated by denaturing PAGE, detected by fluorescence scanning and quantified. Data are the mean and standard error from three independent experiments.

We next investigated whether FPG, the main AP lyase detected in *Arabidopsis* cell extracts [26], is also affected by the sequence context and/or the base opposite the AP site. We performed incision assays with recombinant FPG protein on the different DNA substrates, and the results obtained indicate a clear and consistent preference for AP sites opposite cytosine in all three sequence contexts (Figure 5). In general, the AP lyase activity of FPG was lower at higher DNA concentrations, regardless of the opposite base or the sequence context, suggesting a possible inhibition by substrate. As a result, the preference for C as the orphan base was stronger at the low DNA concentration (Figure 5A). For example, in context C (20 nM), FPG processed about 100% of the substrate containing AP:C after 90 min, compared to 20% substrate with AP:G.

The AP lyase activity of FPG on its preferred target (AP:C) was less efficient in context A compared to contexts B and C. Thus, for 40 nM substrate, only 30% of AP:C lesions were processed at 90 min, in contrast with 70% or 80% in contexts B and C, respectively (Figure 5B). In these same conditions, no clear differences between contexts were detected for AP:G targets. 

We also analysed the AP lyase activity of the native FPG enzyme using cells extracts from ARP deficient plants. As shown in Figure 3, we corroborated that *arp^−/−^* extracts had similar quality and efficiency in uracil DNA repair than WT and *fpg^−/−^* extracts (Figure 3A), and then analysed AP endonuclease and AP lyase activity using an AP site generated by uracil excision (Figure 3B). As expected, AP incision levels of *arp^−/−^* extracts were similar to those of WT extracts, but only in the absence of Mg^2+^. Such Mg^2+^-independent activity was lost in *fpg^−/−^* extracts, demonstrating that *arp^−/−^*-deficient plants only possess AP lyase activity from FPG.

**Figure 6:**
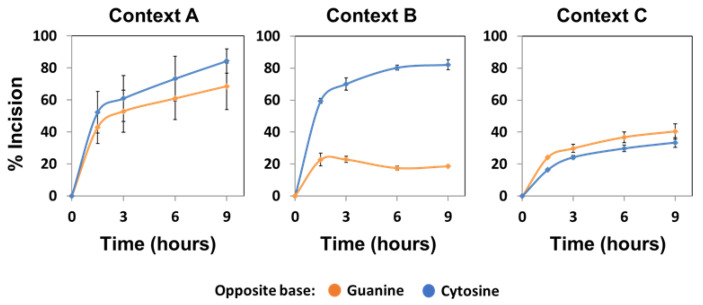
Effect of sequence context and base opposite the abasic site on the AP lyase activity of native FPG. Cell extracts from *arp^−/−^* plants (25 μg) were incubated at 37 °C with 20 nM DNA substrates (contexts A, B or C) containing an AP site opposite guanine (orange) or cytosine (blue). After stabilization with NaBH_4_, the reaction products were separated by denaturing PAGE, detected by fluorescence scanning and quantified. Data are the mean and standard error from three independent experiments.

We then performed AP incision assays with *arp^−/−^* extracts and different DNA substrates. Similarly, with recombinant FPG, the results obtained with native FPG also revealed a higher efficiency processing AP sites opposite cytosine (Figure 6). This preference was more evident with context B, in which 60% of the substrate was processed in 90 min when the opposite base was cytosine, while only 20% was processed when the base opposite the AP site was guanine (Figure 6). With context A, a preference for cytosine as the opposing base was also observed, although less marked (Figure 6). Unlike the results obtained with contexts A and B, with context C we observed the lowest levels of incision and no effect depending on the base opposite the AP site (Figure 6). As for the effect of the sequence context on the AP lyase activity of native FPG, we observed greater differences when the AP site was opposite cytosine. A comparison of E_rel_ values for AP:C substrates revealed a preference for contexts A and B (E_rel_ 0.23 ± 0.01 and 0.32 ± 0.01, respectively) compared to context C (0.07 ± 0.00) (Supplementary Table S2). In contrast, with AP:G targets we observed a preference for context A (0.14 ± 0.01) compared to context C (0.10 ± 0.00) (no reliable E_rel_ value could be estimated for context B) (Supplementary Table S2). Altogether, these results indicate that the main AP lyase from *Arabidopsis* exhibits a preference for abasic sites opposite cytosine, and that its enzymatic activity is modulated by the specific sequence flanking the lesion.

### 2.4. The Major Human AP Endonuclease, APE1, Exhibits a Preference for G as the Orphan Base, but Not in All Sequence Contexts

**Figure 7:**
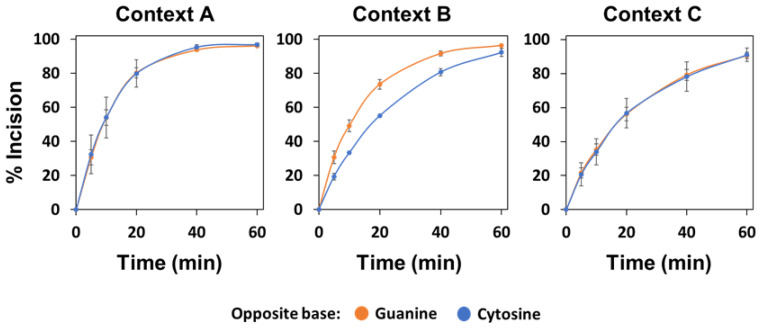
Effect of sequence context and base opposite the abasic site on the AP endonuclease activity of recombinant human APE1. APE1 protein (4 nM) was incubated at 37 °C with 80 nM DNA substrates (contexts A, B or C) containing an AP site opposite guanine (orange) or cytosine (blue). After stabilization with NaBH_4_, reaction products were separated by denaturing PAGE, detected by fluorescence scanning and quantified. Data are the mean and standard error from three independent experiments.

Next, we wondered if the inverse preference for the orphan base exhibited by plant AP endonucleases and AP lyases and the modulatory effect exerted on their activities by the flanking DNA sequence were common features present in other organisms such as humans. We first analysed the enzymatic activity of recombinant and native APE1, the major human AP endonuclease [33], on the different DNA substrates. The recombinant version of human APE1 showed a slight preference for AP sites opposite guanine but only in sequence context B (E_rel_ 8.03 ± 0.06 and 4.67 ± 0.05 for AP:G and AP:C, respectively) (Figure 7, Supplementary Table S3). Regarding the context sequence, and irrespective of the base opposite the abasic site, the highest processing activity was observed with context A (with E_rel_ values close to 9), while the lowest was observed with context C (with E_rel_ values close to 5) (Figure 7). 

**Figure 8:**
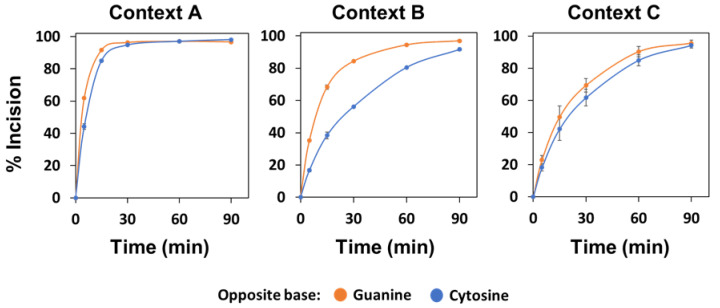
Effect of the sequence context and base opposite to the abasic site on the native AP endonuclease activity. Cell extracts (0.1 μg) from U2OS were incubated at 37 °C with 20 nM DNA substrates (contexts A, B or C). After stabilization with NaBH_4_, the reaction products were separated by denaturing PAGE, detected by fluorescence scanning and quantified. Data are the mean and standard error from three independent experiments.

We also analysed the activity of native human APE1 using U2OS osteosarcoma cell extracts. The results obtained were very similar to those obtained with recombinant APE1 (Figure 8), suggesting that most, if not all, of the native AP endonuclease activity detected in these cell extracts came from APE1. However, in this case, the preference for orphan G was also marginally detectable in contexts A and C (Figure 8 and Supplementary Table S3). 

**Figure 9:**
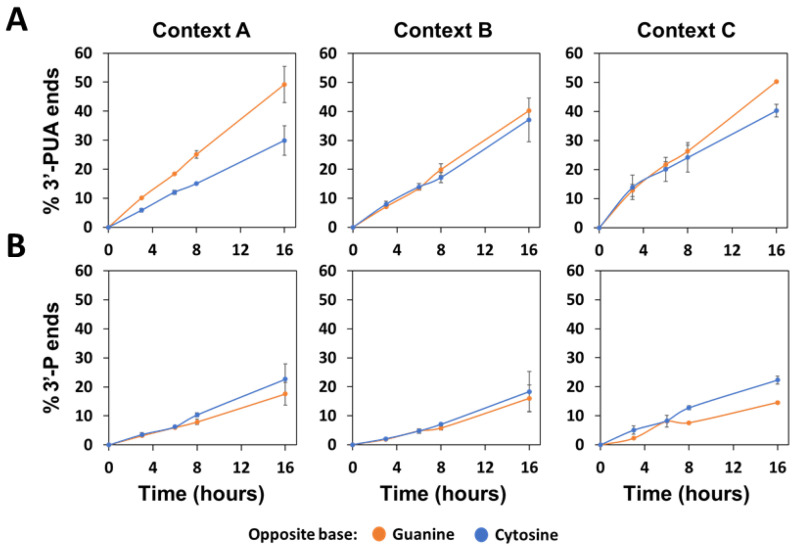
Effect of sequence context and base opposite the abasic site on the native AP lyase activity detected in human cells. Extracts from U2OS cells (20 μg) were incubated at 37 °C with 20 nM DNA substrates (context A, B or C) containing an AP site opposite guanine (orange) or cytosine (blue). After stabilization with NaBH_4_, the reaction products were separated denaturing PAGE, detected by fluorescence scanning and quantified. Graphs show percentage of incised products with 3′-PUA ends (**A**) or 3′-P ends (**B**). Data are the mean and standard error from three independent experiments.

We next examined the native AP lyase activity in U2OS cell extracts. Unlike plant extracts, human extracts exhibited the activity of several AP lyases, some of which generated 3′-PUA ends (NTH1 and OGG1) and others produced 3′-P termini (NEIL1, NEIL2 and NEIL3) [34]. We, therefore, quantitated both types of DNA repair intermediates in the three different sequence contexts. We found that the accumulation of 3′-PUA ends was faster for AP:G than for AP:C targets, whereas the reverse was observed for 3′-P ends (Figure 9). These results suggest that human β-elimination and β, δ-elimination catalysts prefer G and C, respectively, as the orphan base.

## 3. Discussion

All cellular organisms possess both AP endonucleases and AP lyases, two different enzymatic activities able to incise abasic sites, but the factors explaining such an apparently redundant role remain poorly understood. In this work, we tested the hypothesis that the specific sequence surrounding an AP site and/or the identity of the orphan base influences the enzymatic activities of AP endonucleases and AP lyases. We concentrated our study on AP sites arising from C:G pairs located within sequence contexts with different probabilities to be targeted by the DNA methylation machinery. 

Although context-dependent excision has been reported for several DNA glycosylases [35], the effect of the flanking DNA sequence on AP incision remains unexplored. In this work, we found significant differences in AP site incision efficiency by both AP endonucleases and AP lyases in different sequence contexts. However, there was no clear correlation between AP incision efficiency and the expected probability of methylation at the C:G pair in which the AP site arose. For example, the native ARP endonuclease activity on AP:G targets was equally efficient in contexts A and C, in which the expected probability of the lost cytosine to be methylated is low and high, respectively. In comparison, it was significantly lower in context B, with an intermediate probability of methylation. Similarly, the native AP lyase activity of FPG on AP:C targets was significantly higher in context A compared to context C, although the probability of the orphan cytosine to be methylated is very similar in both sequences. Likewise, no correlation with DNA methylation probability was found either for human AP endonuclease or AP lyase activities. 

These results suggest that the capacity of AP endonucleases or AP lyses to incise an AP site arising at a C:G pair is not related to the probability of such a pair to be epigenetically modified. Apparently, the sequence context preference shown by some DNA glycosylases excising frequent lesions arising at CpG sites, such as mismatched U or T [28,29,30], is not present in downstream steps in the BER pathway, such as AP incision. 

In any case, the methylation-independent differences in AP processing efficiency detected between different sequence contexts suggests that the specific sequence surrounding an abasic site influences the efficiency of AP endonucleases and AP lyases. Systematic studies with a large set of DNA substrates will be needed to identify which sequence features influence AP endonuclease and AP lyase activity and the mechanism involved.

An important finding arising from our study was the reverse preference of ARP endonuclease and FPG lyase for the orphan base at AP sites originated from C:G pairs. Whereas ARP favoured G as the estranged base, FPG displayed a preference for abasic sites opposite C. 

The preference of FPG for AP sites opposite C is in agreement with our previous study showing that this enzyme is critical for the excision of AP sites arising from the spontaneous loss of N7-meG [26]. In the present work, the preference of FPG lyase activity for AP:C targets was observed with the recombinant enzyme in all tested sequence contexts, whereas with the native activity in cells extracts it was detected in contexts A and B, but not C. One possible explanation for such a discrepancy is that interacting protein partners present in the cell extract modulate the orphan base preference of FPG in a sequence-dependent manner. On the other hand, it is worth noting that the recombinant protein used in our study is one (FPG1) of seven potential isoforms generated by the alternative splicing of the FPG primary transcript [36]. FPG1 mRNA is expressed in flowers and roots but poorly in leaves, which were the primary material used in our cell extracts. Future work will be needed to analyse whether the preference for AP:C targets is conserved in all FPG isoforms. 

Proteins from the Fpg subfamily are bifunctional DNA glycosylases involved in oxoG repair in both prokaryotes [37] and plants [38]. Given their role in removing oxidized guanine, a preference for C in the complementary strand is not unexpected. However, there are conflicting reports on the efficiency of bacterial Fpg excising oxoG opposite C or G. Thus, one study reported that *E. coli* Fpg processes oxoG:C about 5-fold faster than oxoG:G [39], whereas another report showed about a 10-fold higher activity on oxoG:G than on oxoG:C [40]. On the other hand, *E. coli* Fpg excises N(4),5-dimethylcytosine opposite C, but not opposite G [41]. To our knowledge, no data on the AP lyase activity of prokaryotic Fpg on DNA substrates with different opposite bases have been published.

Unlike plant extracts, in which the only detectable AP lyase activity is that of FPG [26], human cell extracts likely contain a mixture of different AP lyases, making it difficult to detect specific preferences for the orphan base. There are five human bifunctional DNA glycosylases with AP lyase activity NTH1 and OGG1 are β-elimination catalysts and, therefore, generate 3′-PUA ends, whereas NEIL1, NEIL2 and NEIL3 are β, δ-elimination catalysts and generate 3′-P ends [34]. We found that the accumulation of 3′-PUA ends generated by the AP lyase activity of human cell extracts was higher for AP:G than for AP:C targets, while the inverse situation was detected for 3′-P ends. Interestingly, it has been reported that human NTH1 preferentially incises AP sites opposite G [42], which might partially explain the higher accumulation of 3′-PUA incision products that we have detected for AP:G targets. The most likely candidates for the detected 3′-ends are NEIL1, NEIL2 and/or NEIL3, but their opposite base preference as AP lyases, if any, remains unknown. 

Conversely, to the preference for orphan C of *Arabidopsis* FPG AP lyase, we found that ARP endonuclease displayed a significantly higher activity on AP sites opposite G. Whereas the preference of recombinant ARP for AP:G over AP:C was similar among all three sequence contexts, the native ARP activity detected in plant cell extracts showed between a 2-fold and 15-fold higher efficiency on AP:G than on AP:C targets, depending on the specific sequence context. Such a preference for G as the orphan base might partially explain the very poor activity of ARP on AP sites arising from the spontaneous loss of N7-meG [26]. 

Interestingly, and in agreement with our previous results [26], we found that human APE1 endonuclease also displayed some preference for AP sites opposite G, particularly in reactions catalysed by the native AP endonuclease activity detected in cell extracts. Thus, native APE1 displayed between a 1.3-fold and 2.3-fold higher efficiency on AP:G than on AP:C targets, depending on the specific sequence context. The modulatory effect exerted by the sequence flanking the AP site on the opposite base dependence is apparently different for plant and human AP endonucleases. Thus, for ARP, the preference for orphan G was more evident in contexts A and C, but for human APE1 such a preference was greater in context B. 

Conflicting results have been previously published on opposite base effects on APE1 activity. Some studies did not find any detectable influence of the orphan base [43], but others reported a preference for a purine opposite the AP site [44]. Interestingly, one study found that AP sites opposite G were repaired 1.2–4.7-fold more efficiently than AP-sites opposite A in five out of eight different human cell extracts [45]. It is possible that the modulatory effects of the flanking sequence might partially explain such disparities. 

Our results suggest that FPG and ARP specifically interacts with the base opposite the AP site. Interestingly, a structural study with *Lactococcus lactis* Fpg in a complex with an AP site analogue proposed that a pyrimidine as the orphan base contributes to the optimal conformation of the substrate through specific interactions with an Arg residue that is conserved in *Arabidopsis* FPG [46]. Therefore, conserved structural features in Fpg homologs may explain the preference for AP sites opposite C that we have observed with FPG. 

It is also possible that FPG and/or ARP discriminate between conformational differences in AP sites opposite different bases. For example, NMR studies have revealed that the ratio of the different anomeric forms in which an AP site exists may depend upon the identity of the opposite base in the complementary strand [47].

Altogether, our results suggest that, at least in plants, AP endonucleases and AP lyases perform complementary roles in processing AP sites arising at C:G pairs. We propose a model (Supplementary Figure S1) in which FPG AP lyase preferentially targets AP sites arising from a lost guanine that has been either spontaneously released, frequently after alkylation damage, or enzymatically excised, commonly after oxidation damage. In contrast, ARP endonuclease favours AP sites originating from a missing cytosine, most of which arise during BER of uracil. Future studies are needed to determine whether a similar distribution or roles is observed in other organisms and could be related to the evolutionary origin of AP endonucleases and AP lyases.

## 4. Materials and Methods

### 4.1. Plant Material and Cell Extract Preparation

*Arabidopsis arp^−/−^* (SALK_021478) and *fpg^−/−^* (SALK_076932) lines were previously described [26,48].

WT (Col-0) and mutant *Arabidopsis* seeds were surface sterilized and plated on 10 cm Petri dishes containing 25 mL of 0.44% (*w*/*v*) Murashige and Skoog (MS) medium (Sigma, St. Louis, MO, USA) supplemented with 2% (*w*/*v*) sucrose and 0.8% (*w*/*v*) agar, pH 5.8. Plates were incubated under long-day conditions in a growth chamber at 23 °C. After 15 days, seedlings were collected, frozen in liquid nitrogen and stored at 80 °C until use. Whole cell extract were prepared as described [49].

### 4.2. Cell Culture and Cell Extract Preparation

Human Bone Osteosarcoma Epithelial Cells, U2OS, purchased from ATCC (U-2 OS, ATCC^®^ HTB96™), were grown in McCoy’s 5a Medium (Biowest, Nuaillé, France), supplemented with 10% foetal bovine serum (FBS, Biowest) and 1% penicillin/streptomycin (Sigma). Cells were maintained in a humidified atmosphere at 37 °C and 5% CO_2_. After cultures became 80% confluent (usually 4 days), cells were trypsinized and counted. Cell pellets (6 × 10^6^ cells) were collected by centrifugation, washed twice in PBS (Sigma, St. Louis, MO, USA), frozen in liquid nitrogen and stored at −80 °C until use.

Cell pellets were resuspended in lysis buffer (20 mM Tris HCl pH 7.4, 60 mM NaCl, 1 mM DTT, 10% glycerol, 10 μL/mL protease inhibitor Calbiochem^®^ Protease Inhibitor Cocktail Set III, Animal-Free) and lysed by sonication during 3 pulses of 15 s (Bioruptor, Diagenode, Seraing, Belgium). Subsequently, they were centrifuged at 14,000 rpm at 4 °C during 8 min and the supernatant was dialyzed at 4 °C against dialysis buffer (20 mM Tris HCl pH 7.4, 60 mM NaCl, 1 mM EDTA, 1 mM DTT, 17% glycerol) during 2 h and, after buffer change, dialysis continued during 16 h more. Protein concentration was determined by the Bradford assay [50] and the extract was stored in small aliquots at −80 °C. 

### 4.3. Protein Expression and Purification

His-ARP and His-FPG were expressed and purified as previously described [26,51].

### 4.4. Reagents and Enzymes

*Escherichia coli* UDG and human AP endonuclease APE1 were obtained from New England Biolabs (NEB).

### 4.5. DNA Substrates

Oligonucleotides used as DNA substrates (Table 1) were synthesized by IDT and purified by PAGE before use. Double-stranded DNA substrates were prepared by mixing a 5 μM solution of a 5′-fluorescein (Fl)-labelled oligonucleotide with a 10 μM solution of an unlabelled complementary oligonucleotide. Annealing reactions were carried out by heating at 95 °C for 5 min followed by slowly cooling to room temperature.

DNA substrates containing an enzymatic AP site were generated by incubating a DNA duplex containing either a U:G or a U:C mismatch, prepared as described above, with *E. coli* UDG (0.2 U) at 37 °C for 30 min.

### 4.6. DNA Incision Assay

Reactions (50 μL) contained 45 mM Hepes-KOH, pH 7.8, 70 mM KCl, 1 mM DTT, 0.4 mM EDTA, 36 μg BSA, 0.2% glycerol, DNA substrate (20, 40 or 80 nM), the indicated amount of cell extract or protein and, when specified, MgCl_2_ (2 mM) or EDTA (20 mM). After incubation at 37 °C or 30 °C, as specified, reactions were stopped at the indicated time by adding 20 mM EDTA, 0.6% SDS and 0.5 mg·mL^−1^ proteinase K, and mixtures were incubated at 37 °C for 30 min. When indicated, reaction products were stabilized by the addition of freshly prepared sodium borohydride (NaBH_4_; Sigma-Aldrich) to a final concentration of 230 mM and incubated at 0 °C for 30 min. The reaction was buffered with 5 μL of Tris-HCl pH 7.5 and 60 μL of TE (10 mM Tris-HCl pH 8.0, 1 mM EDTA pH 8.0). As a control, a sample without extract was alkaline treated (NaOH, 15 mM) for 10 min at 70 °C to cleave all AP sites present. Subsequently, this sample was buffered with 1.98 μL of Tris-HCl pH 7.5 and 60 μL of TE.

DNA was extracted with phenol:chloroform:isoamyl alcohol (25:24:1) and ethanol-precipitated at −20 °C in the presence of 0.3 mM NaCl and 16 mg·mL^−1^ glycogen. Samples were resuspended in 10 μL of 90% formamide and heated at 95 °C for 5 min. 

Reaction products were separated in a 12% denaturing polyacrylamide gel containing 7 M urea. Labelled DNA was visualized using FLA-5100 imager and analysed using Multigauge software (Fujifilm).

### 4.7. Kinetic Analysis

We used a previously described model used for the kinetic analysis of human thymine DNA glycosylase (TDG) [52] and the *Arabidopsis* ROS1 5-methylcytosine DNA glycosylase [53]. The product concentration (nM) obtained for the different samples and DNA substrates tested were adjusted to the equation [Product] = P_max_ [1 − exp ^(−kt)^] by a non-linear regression analysis performed with SigmaPlot software. In each case, we determined the parameters P_max_ (maximum concentration of processed substrate), T_50_ (time required to reach 50% of the maximum product, P_max_) and E_rel_ (relative processing efficiency, calculated as P_max_/T_50_).

## Figures and Tables

**Table 1:** Oligonucleotides used as substrates.

Name ^a^	DNA Sequence ^b^	Strand ^c^
Fl_UGCG-F	CACGGGATCAATGTGTTCTTTCAGCTCG**U**GCGTCACGCTGACCAGGAATAC	U
UGCG-RG	GTATTCCTGGTCAGCGTGACGC**G**CGAGCTGAAAGAACACATTGATCCCGTG	L
UGCG-RC	GTATTCCTGGTCAGCGTGACGC**C**CGAGCTGAAAGAACACATTGATCCCGTG	L
Fl_CUCG-F	CACGGGATCAATGTGTTCTTTCAGCTCGC**U**CGTCACGCTGACCAGGAATAC	U
CUCG-RG	GTATTCCTGGTCAGCGTGACG**G**GCGAGCTGAAAGAACACATTGATCCCGTG	L
Fl_UCCG-F	CACGGGATCAATGTGTTCTTTCAGCTCG**U**CCGTCACGCTGACCAGGAATAC	U
UCCG-RG	GTATTCCTGGTCAGCGTGACGG**G**CGAGCTGAAAGAACACATTGATCCCGTG	L
UCCG-RC	GTATTCCTGGTCAGCGTGACGG**C**CGAGCTGAAAGAACACATTGATCCCGTG	L
Fl-UGF	TCACGGGATCAATGTGTTCTTTCAGCTC**U**GGTCACGCTGACCAGGAATACC	U
CGR_G	GGTATTCCTGGTCAGCGTGACC**G**GAGCTGAAAGAACACATTGATCCCGTGA	L

^a^ Fl, Fluorescein; ^b^, relevant bases are underlined; ^c^ U, upper; L, lower.

## Supplementary Materials

The following are available online at https://www.mdpi.com/article/10.3390/ijms22168763/s1.
