# Supplementary material for: Complementary Functions of Plant AP Endonucleases and AP Lyases during DNA Repair of Abasic Sites Arising from C:G Base Pairs
Source: Int J Mol Sci. 2021 Aug 16;22(16):8763. doi: 10.3390/ijms22168763 (PMC8395712; doi:10.3390/ijms22168763)
Supplement: Supplementary file 1 [file ijms-22-08763-s001.zip › ijms-1336670-supplementary.pdf]

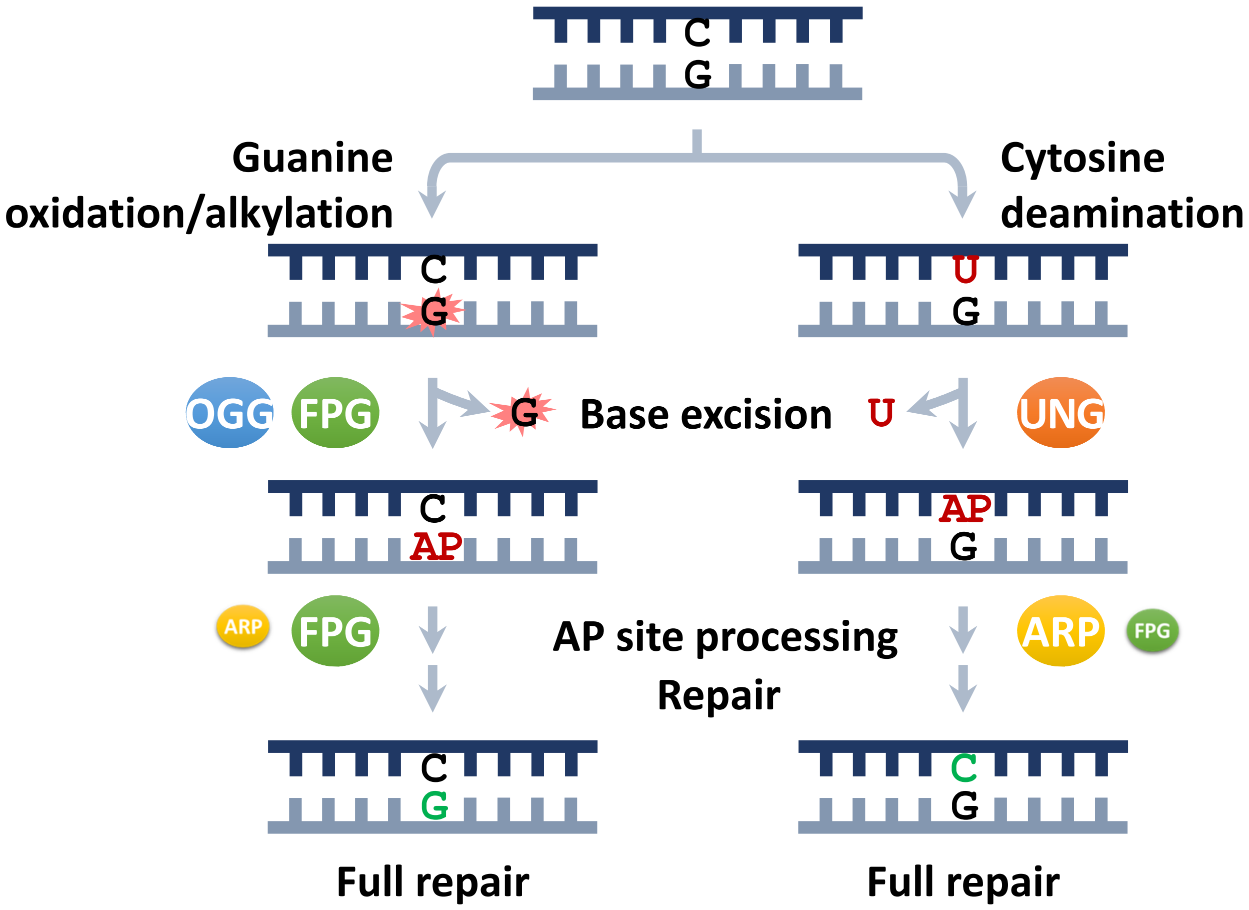

Supplementary Figure S1. Model for repair of AP sites arising from C:G pairs in plants. ARP pref-erentially repaired AP sites arising from cytosine deamination, whereas FPG favoured those derived from guanine oxidation or alkylation. See text for details.

Supplementary Table S1. Probability of methylation of DNA substrates used depending on the orphan base opposite the AP site.

Sequence Context	Orphan Base	Sequence	Probability of Methylation [a]	
			Plants	Humans
A	G	TCG**AP**CCG<br>AGC **G** GGC	Low	Low
B	G	CGC**AP**CGT<br>GCG **G** GCA	Medium	Low
C	G	TCG**AP**GCG<br>AGC **G** CGC	High	High
A	C	TCG**AP**CCG<br>AGC C GGC	Medium	Low
B	C	CGC**AP**CGT<br>GCG C GCA	High	High
C	C	TCG**AP**GCG<br>AGC C CGC	Medium	Low

[a] Probability of cytosine methylation at the underlined position.

Supplementary Table S2. Relative efficiency of native enzymes from *Arabidopsis* on DNA substrates with an AP site in three different sequence contexts and opposite G or C as the orphan base.

Enzyme	Sequence Context	Orphan Base	P_{max} (nM)	T_{50} (min)	E_{rel}
ARP	A	G	19.40 ± 0.11	6.27	3.10 ± 0.02
ARP	A	C	19.01 ± 1.84.	97.63	0.19 ± 0.02
ARP	B	G	19.17 ± 0.28	13.92	1.38 ± 0.02
ARP	B	C	19.21 ± 0.24	25.48	0.75 ± 0.01
ARP	C	G	19.43 ± 0.12	6.66	2.91 ± 0.02
ARP	C	C	18.55 ± 0.76	49.16	0.38 ± 0.02
FPG	A	G	12.37 ± 0.97	88.87	0.14 ± 0.01
FPG	A	C	15.88 ± 0.93	68.63	0.23 ± 0.01
FPG	B	G	n.d. [a]	n.a. [b]	n.a. [b]
FPG	B	C	16.10 ± 0.39	50.23	0.32 ± 0.01
FPG	C	G	7.82 ± 0.33	74.53	0.10 ± 0.00
FPG	C	C	6.65 ± 0.17	94.95	0.07 ± 0.00

[a] n.d.–not determined. [b] n.a.–not applicable.

Supplementary Table S3. Relative efficiency of recombinant or native human APE1 on DNA substrates with an AP site in three different sequence contexts and opposite G or C as the orphan base.

APE1	Sequence Context	Orphan Base	P_{max} (nM)	T_{50} (min)	E_{rel}
Recombinant	A	G	78.11 ± 0.93	8.55	9.14 ± 0.11
Recombinant	A	C	78.70 ± 0.53	8.55	9.21 ± 0.06
Recombinant	B	G	77.95 ± 0.60	9.71	8.03 ± 0.06
Recombinant	B	C	81.16 ± 0.85	17.37	4.67 ± 0.05
Recombinant	C	G	76.84 ± 1.63	15.40	4.99 ± 0.11
Recombinant	C	C	77.49 ± 1.69	15.86	4.89 ± 0.11
Native	A	G	19.32 ± 0.03	3.41	5.67 ± 0.01
Native	A	C	19.54 ± 0.17	5.48	3.56 ± 0.03
Native	B	G	18.98 ± 0.31	8.15	2.33 ± 0.04
Native	B	C	19.18 ± 0.64	21.94	0.87 ± 0.03
Native	C	G	19.20 ± 0.44	14.75	1.30 ± 0.03
Native	C	C	19.38 ± 0.47	19.25	1.01 ± 0.02
